# Volatile biomarkers of Gram-positive bacteria of clinical relevance as a tool for infection diagnosis

**DOI:** 10.1007/s10123-024-00511-z

**Published:** 2024-03-21

**Authors:** Ricardo Rubio-Sánchez, Esperanza Lepe-Balsalobre, Cristina Ubeda, José Antonio Lepe-Jiménez

**Affiliations:** 1 Servicio de Análisis Clínicos, Hospital Universitario de Jerez de la Frontera, Cádiz, Spain; 2 Servicio de Análisis Clínicos, Hospital de Riotinto, Huelva, Spain; 3 Departamento de Nutrición y Bromatología, Toxicología y Medicina Legal, Facultad de Farmacia, Universidad de Sevilla, C/ Profesor García González, 2, 41012 Seville, Spain; 4 Clinical Unit of Infectious Diseases, Microbiology and Preventive Medicine, Infectious Diseases Research Group, Institute of Biomedicine of Seville (IBIS), University of Seville/CSIC/University Hospital Virgen del Rocío, Seville, Spain

**Keywords:** Biomarkers, *Enterococcus faecalis*, *Listeria monocytogenes*, *Staphylococcus aureus*, VOC

## Abstract

**Aim:**

Volatile organic compounds (VOCs) are being studied as potential biomarkers in many infections. Therefore, this study aimed to analyze the volatile profile of three Gram-positive bacteria of clinical relevance to identify potential volatile biomarkers that allow their differentiation.

**Methods and results:**

*L. monocytogenes*, *S. aureus*, and *E. faecalis* clinical isolates were inoculated in a thioglycollate medium until grown. Then, VOCs were extracted by solid-phase microextraction, and the data obtained were subjected to multivariate analysis. According to our results, there was a high production of aldehydes in *E. faecalis*. In the case of alcohols, they only increased in *L. monocytogenes*, while ketones were produced significantly in all three bacteria, mainly due to acetoin. Acids were produced significantly in *E. faecalis* and *L. monocytogenes*.

**Conclusions:**

Potential biomarkers of *L. monocytogenes* could be 1-butanol and 2-methylbutanoic acid. In the case of *E. faecalis*, the VOC most related to its presence was nonanal. Lastly, potential biomarkers of *S. aureus* could be isoamyl butanoate and methionol, although some pyrazines have also been associated with this bacterium.

**Significance and impact of the study:**

The identification of potential biomarkers of these clinically relevant bacteria could open the way for the diagnosis of these infections through the analysis of volatile compounds.

## Introduction

Infections caused by Gram-positive bacteria are a public health problem due to the outbreaks of food poisoning that it can cause and the worrying increase of bacterial resistance to antibiotics that have been taking place in recent years. Among the Gram-positive bacteria involved in outbreaks of food poisoning, the most notable are *Listeria monocytogenes*, *Bacillus cereus*, *Staphylococcus aureus*, *Clostridium perfringens*, and *Clostridium botulinum*. *L. monocytogenes* causes listeriosis, an infection related to eating contaminated food that affects 1600 people and causes 260 deaths each year worldwide (CDC 2024). Listeriosis is the most severe human foodborne disease due to high hospitalization (99%) and mortality (15.6%) rates (Jiménez et al. 2022). In Spain, in 2019, an outbreak of food poisoning by *L. monocytogenes* was reported with 217 infected people and three deaths associated with the industrially produced larded meat with the CC388 clonal complex consumption (Ministerio de Sanidad, Consumo y Bienestar Social, 2019). In recent years, the upward trend in the number of outbreaks by *Listeria* spp., as in the number of hospitalized cases, is worrying (Jiménez et al. 2022).

*S. aureus* also usually causes food poisoning outbreaks by a toxigenic mechanism, giving rise to the sudden appearance of vomiting and diarrhea since preformed enterotoxins are ingested in food (Vila et al. 2009  2008). Between 2019 and 2020, 121 outbreaks of food poisoning by staphylococcal enterotoxins were reported in Europe, affecting almost 1900 people (Jiménez et al. 2022). In addition, methicillin-resistant *S. aureus* (MRSA), as multiresistant *Enterococcus* spp., are increasingly causing nosocomial infections due to the appearance of new MRSA clones and *mec* gene variants that are not detected by the most common molecular systems (Cantón & Ruiz-Garbajosa 2013). The multiresistant bacteria appearance calls for better use of antimicrobials and the application of epidemiological measures, including the detection of carriers, that reduce their transmission.

Detection of pathogens in samples from the environment (mainly foods) and patients usually involves time-consuming growth in selective media, isolation, and biochemical and molecular diagnostic analyses (Reichert-Schwillinsky et al. 2009). Thus, currently, the methodologies used to diagnose diseases have been diversified to a great extent. The search for specific disease biomarkers in non-invasive samples is a trend in several research areas. Specifically, volatile organic compounds (VOCs) are being studied as potential biomarkers in many diseases, such as cancer (Wen et al. 2020) and Alzheimer’s disease (Ubeda et al. 2022). Furthermore, microorganisms can produce species-specific VOCs as a product of their metabolism and could also be helpful for the diagnosis of infection as an odor fingerprinting (Tait et al. 2014). Thus, the VOCs produced by microorganisms have been employed successfully to identify their presence in biological samples with *Helicobacter pylori* (Ulanowska et al. 2011), *Giardia lamblia* (Ubeda et al. 2019), and SARS-CoV-2 (Lamote et al. 2020), among several others. Specifically, the volatilomic profile of *L. monocytogenes* has already been studied in thioglycolate broth (Lepe-Balsalobre et al. 2022), trypticase soy broth (Chen et al. 2017), and milk samples (Tait et al. 2014). Regarding *S. aureus*, the VOCs produced by this bacterium have also been studied in trypticase soy broth (Chen et al. 2017), blood agar media (Gómez-Mejia et al. 2022), and bronchoalveolar lavage fluid (Nasir et al. 2018). This bacterium, like *E. faecalis*, has also been studied in urine sample (Storer et al. 2011) and blood culture samples (Dolch et al. 2012).Therefore, this study aimed to analyze the volatile profile of the *L. monocytogenes* clonal complex responsible for the recent and severe outbreak in Spain (CC388) in comparison with that produced by other Gram-positive bacteria of clinical relevance, such as *S. aureus* and *E. faecalis*, to identify potential volatile biomarkers that allow their differentiation.

## Materials and methods

This study was not submitted for institutional review board approval because it did not include individual patient data.

### Bacterial strains and culture conditions

The study included the clinical isolate of *Listeria monocytogenes* characterized by Multilocus Sequence typing (MLST) belonging to the CC388 clonal complex involved in the epidemic outbreak of 2019 and two clinical isolates of *Staphylococcus aureus* (ATCC 29213) and *Enterococcus faecalis* (ATCC 29212) obtained from the American Type Culture Collection (ATCC).

A 0.5 McFarland dilution was prepared in sterile water from each isolate (plate count, 1.2 × 10^8^ CFU/mL). Then, a technical triplicate 100 μl inoculum (1 × 10^6^ CFU/mL) was transferred to three fluid thioglycollate medium 20-mL tubes (Becton, Dickinson and Company, USA) and incubated at 37°C for 24 h. The positivity was evaluated by bacterial count in Columbia Agar with 5% Sheep Blood (Becton, Dickinson and Company, USA). An uninoculated tube was used as a control. Tubes were stored at − 80 °C until analysis.

### Extraction of volatile compounds

Solid-phase microextraction (SPME) was employed for the VOC extraction. Tubes were defrosted, and 7.5 mL of the media where microorganisms grew was placed in a 20-mL headspace vial joint to 1.5 g of NaCl and 10 μL of internal standard (4-methyl-2-pentanol, 0.75 mg/L). An MPS Autosampler (Gerstel, USA) incubated the vial for 10 min at 45 °C with agitation at 300 rpm. Then, a 2-cm 50/30 μm Carboxen/DVB/PDMS SPME fiber (Supelco, USA) was exposed to the headspace of the vial for 40 min. Afterward, fiber was desorbed in the injector in a splitless mode for 3 min with the transfer line at a temperature of 250 °C. Analyses were performed in an Agilent 8890 GC system coupled to an Agilent 5977B Inert Plus quadrupole mass spectrometer with a Gerstel autosampler (Müllheim an der Ruhr, Germany). The capillary column and flow rate employed were the same as the analysis. The conditions were as follows: the oven temperature program started at 35 °C held for 4 min, followed by an increase to 220 °C at 2.5 °C/min held for 1 min.

For both analyses, electron ionization mass spectra data were recorded from m/z 29–300 in scan mode with an ionization voltage of 70 eV. All data were recorded using an MS ChemStation (Agilent Technologies, USA).

The VOC identification was done by comparing the mass spectra obtained from each molecule with the reference spectra of the NIST 98 software library and the literature data (Pherobase: www.pherobase.com; NIST Mass Spectrometry Data Center: https://webbook.nist.gov; LRI and Odour database: http://www.odour.org.uk/lriindex.html). When only the software identification was possible, it was treated as tentatively identified. The data showed in this work were expressed as the relative area concerning 4-methyl-2-pentanol (internal standard). The relative concentration was calculated by dividing the peak area of the target ion of each compound by the peak area of the target ion of the internal standard.

### Statistical analysis

The data obtained from the SPME/GC/MS determination were subjected to multivariate analysis performing principal component analysis (PCA) with MetaboAnalyst (Canada).

## Results

### Volatile profile characterization of L. monocytogenes (CC388), E. faecalis, and S. aureus

The employment of the SPME technique allowed the identification of 78 VOCs present in thioglycollate media after the growth of the bacteria. It was found that the control medium, without bacteria, presented several volatile compounds (Table 1). They were not subtracted from the bacterial culture samples because it is also interesting to observe the decrease in the amount of these compounds in the media since it could be due to volatilization but also to their consumption by these pathogens, which would provide interesting information to understand their metabolism. The metabolic pattern of each microorganism gave rise to different amounts of VOCs of each chemical family found. This first approach shows that the VOC pattern observed in *L. monocytogenes* is closer to that of *E. faecalis* than that of *S. aureus*.

**Table 1:** Relative area ranges and linear retention index (LRI) of the volatile organic compounds (VOCs) identified in the thioglycollate media

		LRI	ID	Control	*L. monocytogenes*	*E. faecalis*	*S. aureus*
1	ALDEHYDES						
2	3-Methylbutanal	939	A	34.7 ± 10.1b	39.8 ± 8.7b	42.8 ± 3.1b	12.8 ± 3.1a
3	Nonanal	1382	A	1.32 ± 0.18a	1.66 ± 0.35a	14.2 ± 0.8b	1.45 ± 0.22a
4	3-(methylthio)propionaldehyde	1455	A	1.82 ± 0.34b	1.89 ± 0.46b	2.31 ± 0.34b	NDa
5	Benzaldehyde	1510	A	14.2 ± 2.3a	46.2 ± 13.8b	77.6 ± 19.3c	11.2 ± 2.9a
6	5-Methyl-2-thiophenecarboxaldehyde	1742	A	2.44 ± 0.30a	15.7 ± 3.7b	26.4 ± 3.5c	6.53 ± 1.03a
7	2,5-thiophenedicarboxaldehyde	1798	A	20.7 ± 2.9a	40.2 ± 9.7bc	46.8 ± 1.3c	30.3 ± 5.9ab
8	2-Phenyl-2-butenal	1936	A	0.202 ± 0.029a	0.739 ± 0.222bc	1.15 ± 0.42c	0.286 ± 0.059ab
9	ALCOHOLS						
10	Ethanol	958	A	221 ± 20ab	260 ± 17b	189 ± 20a	230 ± 20ab
11	1-Butanol	1151	A	11.9 ± 0.3a	120 ± 12b	22.6 ± 0.5a	14.2 ± 2.2a
12	2-Methyl-1-butanol	1195	A	NDa	5.88 ± 0.45c	2.26 ± 0.26b	NDa
13	3-Methyl-1-butanol	1198	A	NDa	21.3 ± 1.4b	19.7 ± 1.9b	NDa
14	5-Methyl-3-hexanol	1231	B	5.94 ± 0.82a	6.57 ± 0.28a	6.17 ± 0.45a	6.48 ± 0.63a
15	6-Methyl-2-heptanol	1380	A	0.525 ± 0.078a	0.578 ± 0.125a	0.915 ± 0.035b	0.581 ± 0.006a
16	3,6-Dimethyl-3-octanol	1434	B	0.753 ± 0.018a	0.788 ± 0.115a	0.778 ± 0.078a	0.748 ± 0.105a
17	1-Heptanol	1459	A	0.961 ± 0.161a	1.70 ± 0.23b	2.16 ± 0.13c	1.07 ± 0.09a
18	Dihydromyrcenol	1473	A	0.673 ± 0.122a	0.932 ± 0.269a	0.868 ± 0.174a	0.958 ± 0.160a
19	5-Methyl-1-heptanol	1535	B	0.382 ± 0.054a	0.405 ± 0.028a	0.435 ± 0.067a	0.348 ± 0.057a
20	1-Octanol	1566	A	2.29 ± 0.56a	3.16 ± 0.27bc	3.39 ± 0.31c	2.44 ± 0.32ab
21	2-Decanol	1586	A	0.554 ± 0.092a	0.742 ± 0.153b	0.973 ± 0.071c	0.535 ± 0.039a
22	Levomenthol	1638	A	0.290 ± 0.097b	0.346 ± 0.013b	NDa	0.395 ± 0.121b
23	1-Nonanol	1660	A	1.57 ± 0.28a	3.79 ± 0.75b	4.69 ± 0.36b	1.43 ± 0.09a
24	2-Propyl-1-heptanol	1666	B	0.289 ± 0.043a	0.361 ± 0.067a	0.330 ± 0.014a	0.306 ± 0.023a
25	4-Butoxybutan-1-ol	1704	B	0.086 ± 0.012a	1.06 ± 0.34b	0.906 ± 0.283b	0.172 ± 0.046a
26	2-Undecanol	1725	A	0.316 ± 0.066a	0.535 ± 0.134b	1.55 ± 0.18c	0.277 ± 0.008a
27	Methionol	1728	A	1.08 ± 0.22a	0.821 ± 0.171a	0.733 ± 0.119a	2.80 ± 0.40b
28	1-Decanol	1772	A	1.07 ± 0.18a	1.42 ± 0.12ab	1.72 ± 0.42b	1.19 ± 0.10a
29	3-Acetyl-2.5-dimethylthiophene	1826	A	0.170 ± 0.038a	0.988 ± 0.255ab	1.26 ± 0.23b	0.496 ± 0.142ab
30	3-Methyl-4-(methylthio)phenol	1866	B	1.02 ± 0.09a	1.37 ± 0.40b	1.61 ± 0.13b	1.44 ± 0.24a
31	2-Phenylethanol	1826	A	1.30 ± 0.15a	3.99 ± 0.62c	2.50 ± 0.07b	1.58 ± 0.28a
32	1-Dodecanol	1980	A	2.05 ± 0.30b	1.61 ± 0.05a	1.14 ± 0.13a	1.62 ± 0.19a
33	3-Methylphenol	2080	A	0.203 ± 0.026b	NDa	NDa	0.223 ± 0.048b
34	1-Hexadecanol	2165	A	0.475 ± 0.071a	0.463 ± 0.116a	0.308 ± 0.051a	0.379 ± 0.118a
35	KETONES						
36	Methyl isobutyl ketone	999	A	37.2 ± 3.6b	24.6 ± 2.7a	21.5 ± 2.6a	26.2 ± 4.8a
37	2,6-Dimethyl-4-heptanone	1168	A	11.7 ± 1.1a	13.7 ± 0.9a	13.1 ± 0.7a	11.5 ± 1.1a
38	Acetoin	1271	A	1.72 ± 0.41a	352 ± 13c	449 ± 32d	130 ± 12b
39	Hydroxyacetona	1284	A	3.72 ± 0.89a	5.93 ± 1.31b	5.76 ± 0.78b	5.72 ± 0.66b
40	2-Decanone	1487	A	0.618 ± 0.080b	0.558 ± 0.085b	NDa	0.553 ± 0.041b
41	Acetophenone	1635	A	1.95 ± 0.62a	2.23 ± 0.51a	2.44 ± 0.38a	2.11 ± 0.44a
42	2,4,4-Trimethyl-3-(3-methylbutyl)cyclohex-2-enone	1733	A	22.4 ± 4.8a	34.6 ± 6.3b	32.8 ± 8.2ab	26.4 ± 5.9ab
43	Geranylacetone	1852	A	0.574 ± 0.136a	0.793 ± 0.119a	0.645 ± 0.028a	0.734 ± 0.133a
44	2-Acetylpyrrole	1969	A	0.700 ± 0.047a	0.857 ± 0.054ab	0.888 ± 0.024b	0.890 ± 0.026b
45	PYRAZINES						
46	Pyrazine	1168	A	33.3 ± 8.7a	31.9 ± 9.3a	28.9 ± 3.5a	33.1 ± 4.6a
47	2-Methylpyrazine	1253	A	25.4 ± 5.5a	26.1 ± 5.8a	26.7 ± 1.6a	28.7 ± 8.2a
48	2,5-Dimethylpyrazine	1320	A	9.37 ± 2.47a	13.6 ± 4.1a	13.2 ± 2.2a	15.4 ± 2.4a
49	2-Ethylpyrazine	1328	A	16.6 ± 2.7a	16.7 ± 2.9a	16.4 ± 1.2a	21.4 ± 5.1a
50	2-Ethyl-6-methylpyrazine	1386	A	7.43 ± 1.02a	8.22 ± 0.77a	8.54 ± 0.61a	9.85 ± 2.26a
51	2,3,5-Trimethylpyrazine	1403	A	5.72 ± 1.07a	7.28 ± 2.13a	7.23 ± 0.75a	9.07 ± 1.61a
52	3-Ethyl-2,5-dimethylpyrazine	1444	A	21.3 ± 3.2a	21.1 ± 2.9ab	18.6 ± 3.5a	33.9 ± 8.5b
53	2,6-Diethylpyrazine	1463	A	2.26 ± 0.53a	2.81 ± 0.75a	2.75 ± 0.37a	3.43 ± 0.83a
54	2,3,5,6-Tetramethylpyrazine	1480	A	0.810 ± 0.247a	0.733 ± 0.104a	0.671 ± 0.044a	1.03 ± 0.24a
55	2,3,5-Trimethyl-6-ethylpyrazine	1518	A	2.29 ± 0.13b	1.89 ± 0.13ab	1.67 ± 0.12a	4.38 ± 0.34c
56	2,5-Dimethyl-3-isobutylpyrazine	1530	B	0.772 ± 0.103a	0.751 ± 0.123a	0.767 ± 0.073a	1.49 ± 0.08b
57	2,5-Dimethyl-3-butylpyrazine	1574	A	2.16 ± 0.26a	2.29 ± 0.34a	2.33 ± 0.09a	3.59 ± 0.26b
58	2-Isoamylpyrazine	1585	B	0.657 ± 0.197a	0.744 ± 0.205a	0.715 ± 0.078a	0.983 ± 0.235a
59	2,5-Dimethyl-3-isoamylpyrazine	1656	A	14.4 ± 2.4a	13.3 ± 2.5a	13.1 ± 1.6a	32.5 ± 2.7b
60	ESTERS						
61	Isoamyl butyrate	1247	A	0.495 ± 0.042a	0.265 ± 0.073a	0.377 ± 0.037a	7.61 ± 2.51b
62	Isononyl acetate	1392	B	0.491 ± 0.122a	0.825 ± 0.377a	0.310 ± 0.069a	0.549 ± 0.027a
63	Ethyl octanoate	1422	A	0.821 ± 0.026a	1.29 ± 0.16c	1.09 ± 0.08bc	0.912 ± 0.061ab
64	Methyl decanoate	1588	A	NDa	NDa	NDa	0.736 ± 0.173b
65	Furfuryl thioacetate	1759	A	NDa	2.34 ± 0.79b	2.37 ± 0.50b	NDa
66	Isopropyl dodecanoate	1818	A	0.186 ± 0.021c	0.120 ± 0.012ab	0.105 ± 0.016a	0.141 ± 0.022b
67	2,2-Dimethyl-1-(2-hydroxy-1-methylethyl)propyl 2-methylpropanoate	1881	A	47.9 ± 5.8b	34.7 ± 10.6a	26.1 ± 5.9a	55.1 ± 5.5b
68	ACIDS						
69	Acetic acid	1450	A	NDa	119 ± 6b	239 ± 12c	NDa
70	Propanoic acid	1539	A	NDa	5.38 ± 1.03b	6.20 ± 0.09b	NDa
71	Butyric acid	1600	A	NDa	129 ± 13b	133 ± 5b	1.41 ± 0.54a
72	2-Methylbutanoic acid	1676	B	NDa	41.8 ± 3.7b	NDa	NDa
73	Isobutyric acid	1582	A	NDa	25.5 ± 1.1b	24.3 ± 0.4b	NDa
74	Hexanoic acid	1872	A	NDa	78.1 ± 2.4b	94.7 ± 10.4b	1.57 ± 0.23a
75	Octanoic acid	2073	A	NDa	3.09 ± 0.43a	2.35 ± 0.38a	2.26 ± 0.51a
76	Nonanoic acid	2190	A	NDa	2.16 ± 0.57b	NDa	2.06 ± 0.75a
	OTHER NITROGEN AND SULFUR COMPOUNDS						
77	2,4-Dimethylthiazole	1277	A	1.35 ± 0.34b	1.06 ± 0.19ab	0.513 ± 0.102a	1.29 ± 0.17ab
78	Pyrrole	1501	A	0.942 ± 0.085b	NDa	NDa	NDa
79	2-Propyltetrahydrothiophene	1592	A	NDa	1.80 ± 0.49b	NDa	4.52 ± 0.45c
80	2-Acetylthiazole	1629	A	3.84 ± 1.11a	11.8 ± 1.3b	13.7 ± 1.1c	3.88 ± 0.67a
81	2-Acetylthiophene	1776	B	2.48 ± 0.36a	2.61 ± 0.49a	2.75 ± 0.16a	3.15 ± 0.99a
82	3-Methyl-2-thiophenecarboxaldehyde	1807	A	2.82 ± 0.59a	4.48 ± 0.68b	5.55 ± 0.29c	4.69 ± 0.57bc
83	2-Methylthieno[2,3-b]thiophene	1897	B	13.1 ± 3.2a	13.2 ± 4.4a	14.7 ± 3.5a	19.8 ± 5.1a
84	Toluene-3,4-dithiol	1986	B	15.5 ± 2.8a	17.1 ± 1.3a	17.2 ± 3.8a	19.3 ± 6.3a

Results (average ± SD) are expressed as peak area/100. *ND* not detected, *LRI* linear retention index. Values with different superscript letter indicate statistically significant differences by ANOVA Tukey test (*p* < 0.05). ID: reliability of identification. A: mass spectrum agreed with mass spectral data base and LRI agreed with the literature data (Pherobase: www.pherobase.com; NIST Mass Spectrometry Data Center: https://webbook.nist.gov/; LRI and Odour database: http://www.odour.org.uk/lriindex.html). B: mass spectrum agreed with mass spectral database, but there is no LRI in a polar column reported in the literature. C: mass spectrum agreed with mass spectral data base R.Match ˃ 800 but not with LRI in the literature. Numbers for each compound from the first column correspond to the numeration of Fig. 2

**Fig. 1:**
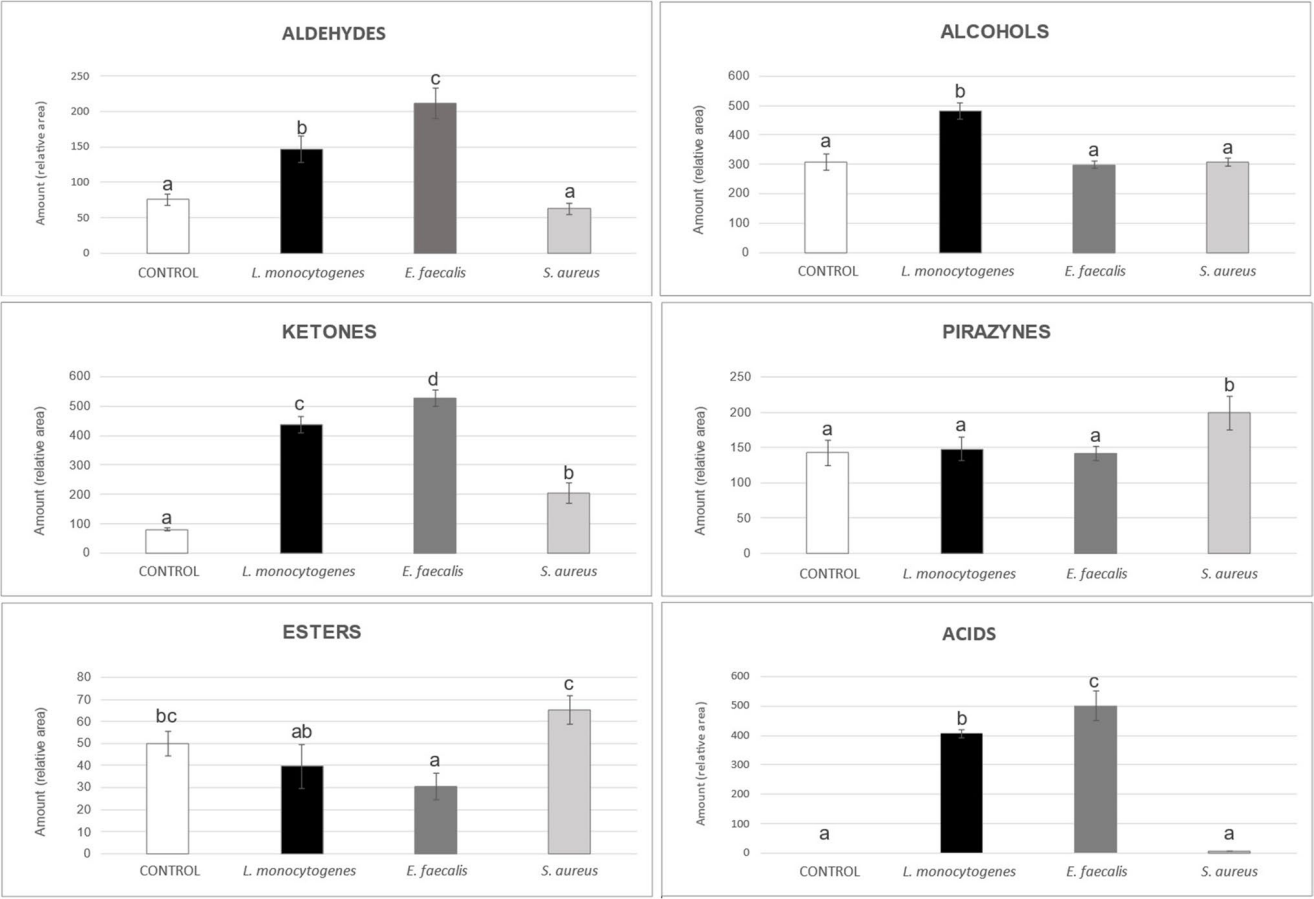
Total volatile amounts of the main chemical families determined in thioglycollate culture medium from *L. monocytogenes* CC388, *E. faecalis*, and *S. aureus*. Different letters in the bars indicate differences between the samples (*p* < 0.05) (Tukey test)

Thus, as can be observed in Fig. 1, in the culture of *S. aureus*, the aldehydes had decreased slightly concerning the control. In contrast, the other two bacteria showed significant aldehyde production, with *E. faecalis* being the most productive. In this study, *L. monocytogenes* was characterized by the production of alcohols, unlike *E. faecalis* and *S. aureus*, which did not vary their total amount concerning the control. Our results showed that all bacteria produce ketones, mainly due to acetoin (Table 1). Esters and compounds derived from pyrazine were also determined, detecting most of them in the control medium.

Volatile acids were the only chemical group not present in the control medium determined with SPME–GC–MS. *E. faecalis*, followed by *L. monocytogenes*, exhibited a remarkable production of volatile acids (Fig. 1). The production of volatile acids by *S. aureus* was significantly lower than the amounts detected in the other bacteria studied. The main difference relied on the short-chain fatty acid (SCFA) production. Thus, acetic, propanoic, and 2-methyl butanoic acids were not detected in the media of *S. aureus*. In addition, butanoic and hexanoic acids were determined in significantly lower amounts than in the *E. faecalis* and *L. monocytogenes* media.

### Multivariate analysis: principal component analysis

**Fig. 2:**
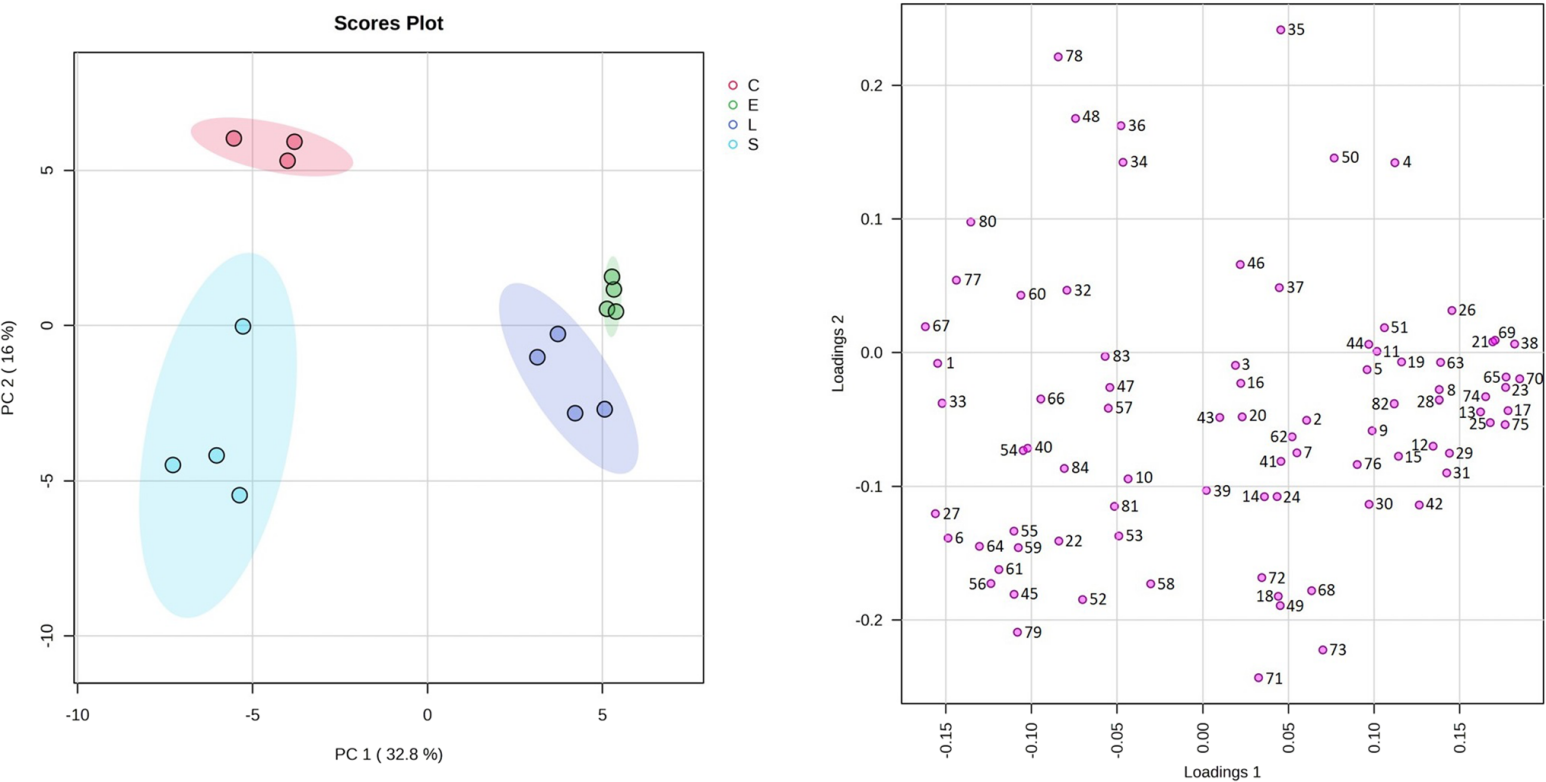
Data scores and loading biplot on the plane of the first two principal components (PC1 against PC2) of the volatiles from *L. monocytogenes* CC388, *E. faecalis*, and *S. aureus*. Numeration of loadings correspond to each compound from the first column of Table 1

**Fig. 3:**
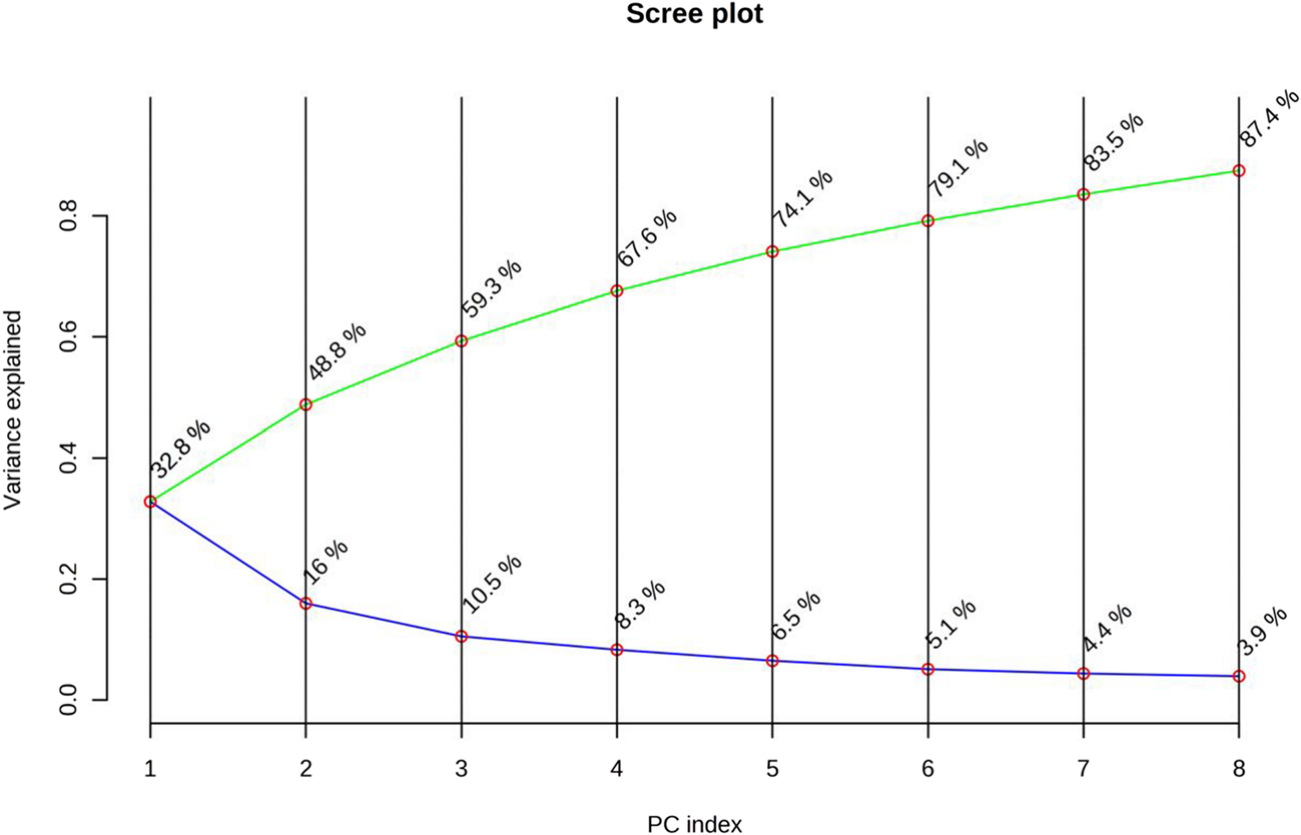
Principal components (PCs) determined by principal component analysis (PCA) that explain different percentages of the total variance

A PCA including all the VOCs and the total sum of each chemical group was performed, as shown in Fig. 2 (84 variables), to interpret the results obtained from the volatile profile comparison of *L. monocytogenes*, *E. faecalis*, and *S. aureus*. The analysis determined eight principal components (PCs) that explained 87.4% of the total variance, with PC1 and PC2 accounting for 48.8% of the accumulated variance (Fig. 3) and permitting a significant separation of the samples. The location in the plane of the samples corroborates that the thioglycollate medium employed for the growth of these bacteria gives rise to similar volatile profiles for *L. monocytogenes* and *E. faecalis*, being different to the amounts and types of volatile metabolites resulting for *S. aureus* growth. Furthermore, it can be observed that acids were clearly correlated with *E. faecalis* and *L. monocytogenes* (Fig. 2).

## Discussion

Although both *E. faecalis* and *L. monocytogenes* produce aldehydes, it is interesting in the first bacterium since it has been little studied. Monedeiro et al. (2021) observed that the volatile profile pattern of *E. faecalis* was the most common, as it had few unique VOCs compared to *Escherichia coli*, *Klebsiella pneumoniae*, and *Proteus mirabilis*. It can be seen in this study that *E. faecalis* significantly produces benzaldehyde and nonanal, being the only bacterium that generates the latter aldehyde (Table 1), so nonanal could be a potential biomarker of its presence in the media.

The highest production of alcohols in *L. monocytogenes* was mainly due to 1-butanol (Table 1), a short-chain alcohol that was significantly increased in all clonal complexes of *L. monocytogenes* (Lepe-Balsalobre et al. 2022). This result contrasts with the findings of Yu et al. (2015) who did not observe 1-butanol among the VOCs produced by *L. monocytogenes* cultured in TSB. This fact might be due to the culture medium, described as one of the main factors defining the VOCs produced by a microorganism (Tait et al. 2014; Sharma et al. 2017). Isoamyl alcohols (2-methyl-1-butanol and 3-methyl-1-butanol) were only produced by *L. monocytogenes* and *E. faecalis*, again reflecting the proximity between their metabolic systems. This fact agrees with D’Angelo et al. (2020) on another bacterium of the same genus, *Enterococcus faecium*, where they observed that leucine was converted to the corresponding keto acid before being decarboxylated to 3-methylbutanal, which could later be reduced to 3-methyl-1-butanol or oxidized to 3-methylbutanoic acid.

The amount of methionol found in the culture medium of *S. aureus* was significantly higher compared to the other microorganisms. This increase, together with the disappearance of 3-(methylthio)propionaldehyde (methional) in the case of *S. aureus*, points to a reduction of 3-(methylthio)propionaldehyde to methionol by the alcohol dehydrogenase activity of this bacteria to metabolize the amino acid methionine. This enzymatic activity has been previously described in other bacteria, such as *Oenococcus oeni* (Vallet et al. 2009).

As mentioned above, the increase of ketones was mainly due to acetoin. The production of this compound by some microorganisms has been widely described through the acid-mixed fermentation pathway of pyruvate. Chen et al. (2017) pointed out that this ketone production indicated the presence of *L. monocytogenes* and *S. aureus* compared to Gram-negative bacteria in the TSB medium. Specifically, Yu et al. (2015) stated that acetoin was the key-compound discriminating samples inoculated with *L. monocytogenes* with an electronic nose. In our study, the highest amount of acetoin in the culture medium was reached by *E. faecalis*, followed by *L. monocytogenes* and *S. aureus*. This fact does not agree with that published by Filipiak et al. (2012), who reported that acetoin meets all the requirements to be a perfect biomarker of *S. aureus* and shows the great need for more research in this field due to the strong dependence on media and comparison with other bacteria. The acetoin formation causes these bacteria to test positive in the Voges Proskauer test, determining their ability to produce this compound from glucose by butanediol fermentation. This biochemical test is important for the identification of *L. monocytogenes* and useful as an indicator of its aerobic growth since acetoin is not produced under anaerobic conditions (Romick et al. 1996).

The amounts of pyrazine and compounds derived from it were very similar in *L. monocytogenes* and the control medium, unlike the results obtained by extracting with dichloromethane (Lepe-Balsalobre et al. 2022). This fact could be due to the different extraction methods since, in the study of the *Listeria* clonal complexes, the extraction was carried out with a polar solvent, which is a more aggressive technique and extracts volatile compounds, but also a lot of non-volatile molecules. Also, the extraction performed in the present study was at 45°C for 50 min, while in Lepe et al. (2022) it was done at room temperature, and maybe the extraction and/or formation of these compounds could be favored by high temperatures. In contrast, these compounds were higher in the case of *S. aureus*, mainly due to the increase of 2,5-dimethyl-3-isoamylpyrazine. This compound has been previously described in food matrices contaminated with *S. aureus* (Fang et al. 2021).

Regarding the esters, due to their lability to acid hydrolysis and evaporation, the apparent consumption by *L. monocytogenes* and *E. faecalis* was probably not due to their metabolism (Fig. 1). However, the data point to a production of esters by *S. aureus*, especially isoamyl butyrate and methyl decanoate (Table 1). The production of esters by *S. aureus* has already been described (Filipiak et al. 2012), but not that of these VOCs in particular, which seem to be biomarkers of the presence of this pathogen in this medium.

The excretion of volatile acids by *E. faecalis* has already been reported in milk (Delgado et al. 2002). However, the variety of acids determined in this study has not been described for *L. monocytogenes.* The volatile acid profile revealed that 2-methylbutanoic acid could be a potential biomarker of *L. monocytogenes* presence (Table 1). SCFAs such as acetic, butanoic, and hexanoic acids are commonly associated with anaerobic metabolism. Thus, *L. monocytogenes* shows a fermentative metabolism due to its beta-d-glucosidase activity and generates acids and diacetyl groups from sugars such as glucose. A large number of SCFAs have been described in various intestinal diseases, such as that caused by *L. monocytogenes* (Ishiguro et al. 2017; Di Cagno et al. 2009). However, it has also been reported that fatty acids in *Listeria* spp. have an important role in resistance against peptidoglycan hydrolases and regulation of virulence (Sun et al. 2012), so they could be involved in the strong pathogenicity and virulence that the CC388 clonal complex presented in the Spanish outbreak in 2019.

Some studies have reported the intolerance of *S. aureus* to SCFAs because they delay and even suppress their growth (Fletcher et al. 2022), and, therefore, this could explain the low contents found in their media. However, 3-methylbutanoic acid that has been previously reported as a unique volatile compound produced by *S. aureus* grown in TSB (Chen et al. 2017) and Brain Heart Infusion (BHI) broth (Tait et al. 2014) was not found in our experiments.

Multiple growth experiments in defined minimal media have shown that most *L. monocytogenes* strains require supplementation of the sulfur amino acids methionine and cysteine for growth. This need is explained by the fact that most *L. monocytogenes* genomes do not express the genes responsible for the sulfate reduction to sulfide, which is subsequently condensed with O-acetylserine to form cysteine (Sauer et al. 2019). The thioglycollate medium contains l-cystine, a dimer of two cysteines linked at their thiol functional groups through a disulfide bond. Some nitrogen and sulfur compounds, such as pyrrole and 2,4-dimethylthiazole, are probably consumed by bacteria. Others, such as 3 methyl-2-thiophenecarboxaldehyde, could be produced from sulfur sources through cysteine consumption (Walker and Schmitt-Kopplin 2021) or formed from the substrate through the Maillard reaction (cysteine + glucose) due to the heating process for 50 min at 45°C during extraction (Umano et al. 1995).

In conclusion, the volatile pattern in the thioglycollate medium observed in *L. monocytogenes* is more similar to that of *E. faecalis* than that of *S. aureus*. Potential biomarkers of *L. monocytogenes* in this medium could be 1-butanol and 2-methylbutanoic acid. In contrast, in the case of *E. faecalis*, the VOC most related to its presence and, therefore, a potential volatile biomarker could be nonanal. Lastly, potential biomarkers of *S. aureus* are isoamyl butanoate and methionol, although some pyrazines have also been associated with this bacterium.

The detection of these volatile biomarkers reveals the possible presence of a specific microorganism and opens the path for future research in biological samples. However, this is a first approach because biological samples contain, in most cases, multiple bacteria that interact with each other, being able to modify the emission of VOCs that they produce when isolated. Therefore, the next research will be focused on the confirmation using biological samples.

## Data Availability

All data can be provided by the corresponding author upon request.
